# Estimates of visual impairment and its causes from the National Eye Survey in Malaysia (NESII)

**DOI:** 10.1371/journal.pone.0198799

**Published:** 2018-06-26

**Authors:** Fiona L. M. Chew, Mohamad Aziz Salowi, Zuraidah Mustari, Mohd Aziz Husni, Elias Hussein, Tassha Hilda Adnan, Nor Fariza Ngah, Hans Limburg, Pik-Pin Goh

**Affiliations:** 1 Department of Ophthalmology, Hospital Selayang, Batu Caves, Selangor, Malaysia; 2 Faculty of Medicine, Universiti Sultan Zainal Abidin, Kuala Terengganu, Terengganu, Malaysia; 3 Department of Ophthalmology, Hospital Sultanah Nur Zahirah, Kuala Terengganu, Terengganu, Malaysia; 4 Clinical Research Centre, Hospital Kuala Lumpur, Kuala Lumpur, Wilayah Persekutuan, Malaysia; 5 Department of Ophthalmology, Hospital Shah Alam, Shah Alam, Selangor, Malaysia; 6 Health Information Services, Grootebroek, The Netherlands; LV Prasad Eye Institute, INDIA

## Abstract

**Background:**

Population-based data on prevalence, causes of blindness and extent of ophthalmological coverage is required for efficient implementation and evaluation of ocular health programs. In view of the scarcity of prevalence data for visual impairment and blindness in Malaysia, this study aims to estimate the prevalence and causes of visual impairment (VI) in the elderly, using Rapid Assessment of Avoidable Blindness (RAAB) survey technique.

**Methods:**

Malaysia was divided into six regions, with each region consisting of 50 clusters. Multistage cluster sampling method was used and each cluster contained 50 residents aged 50 years and above. Eligible subjects were interviewed and pertinent demographic details, barriers to cataract surgery, medical and ocular history was noted. Subjects had visual acuity assessment with tumbling ‘E’ Snellen optotypes and ocular examination with direct ophthalmoscope. The primary cause of VI was documented. Results were calculated for individual zones and weighted average was used to obtain overall prevalence for the country. Inter-regional and overall prevalence for blindness, severe VI and moderate VI were determined. Causes of VI, cataract surgical coverage and barriers to cataract surgery were assessed.

**Results:**

A total of 15,000 subjects were examined with a response rate of 95.3%. The age and gender-adjusted prevalence of blindness, severe visual impairment and moderate visual impairment were 1.2% (95% Confidence Interval: 1.0–1.4%), 1.0% (95%CI: 0.8–1.2%) and 5.9% (5.3–6.5%) respectively. Untreated cataract (58.6%), diabetic retinopathy (10.4%) and glaucoma (6.6%) were the commonest causes of blindness. Overall, 86.3% of the causes of blindness were avoidable. Cataract surgical coverage (CSC) in persons for blindness, severe visual impairment and moderate visual impairment was 90%, 86% and 66% respectively.

**Conclusion:**

Increased patient education and further expansion of ophthalmological services are required to reduce avoidable blindness even further in Malaysia.

## Introduction

Visual impairment drastically impacts a person’s life, giving rise to functional and psychological issues. Widespread visual dysfunction reduces national economic growth, decreases productivity and increases healthcare costs [1]. Treatment of avoidable causes of blindness, especially cataract, has shown to improve quality of life, enhance overall health, alleviate poverty and elevate the economic status of a community [2–4]. The efficient implementation and evaluation of ocular health programs require population-based data on prevalence, causes of blindness and extent of ophthalmological coverage.

The Malaysian National Eye Survey (NES I) in 1996 reported the prevalence of blindness and low vision in Malaysia to be 0.29% and 2.44% respectively in all ages [5]. Cataract was the major cause of bilateral blindness accounting for 39% of total estimated cases. A report using Data Development Analysis noted most public ophthalmological centers could increase output with existing capacity [6]. In view of the scarcity of current prevalence data for visual impairment and blindness, we embarked on the National Eye Survey II (NES II) using Rapid Assessment of Avoidable Blindness (RAAB) methodology to estimate the prevalence of visual impairment among the elderly in Malaysia.

## Material and methods

**Fig 1:**
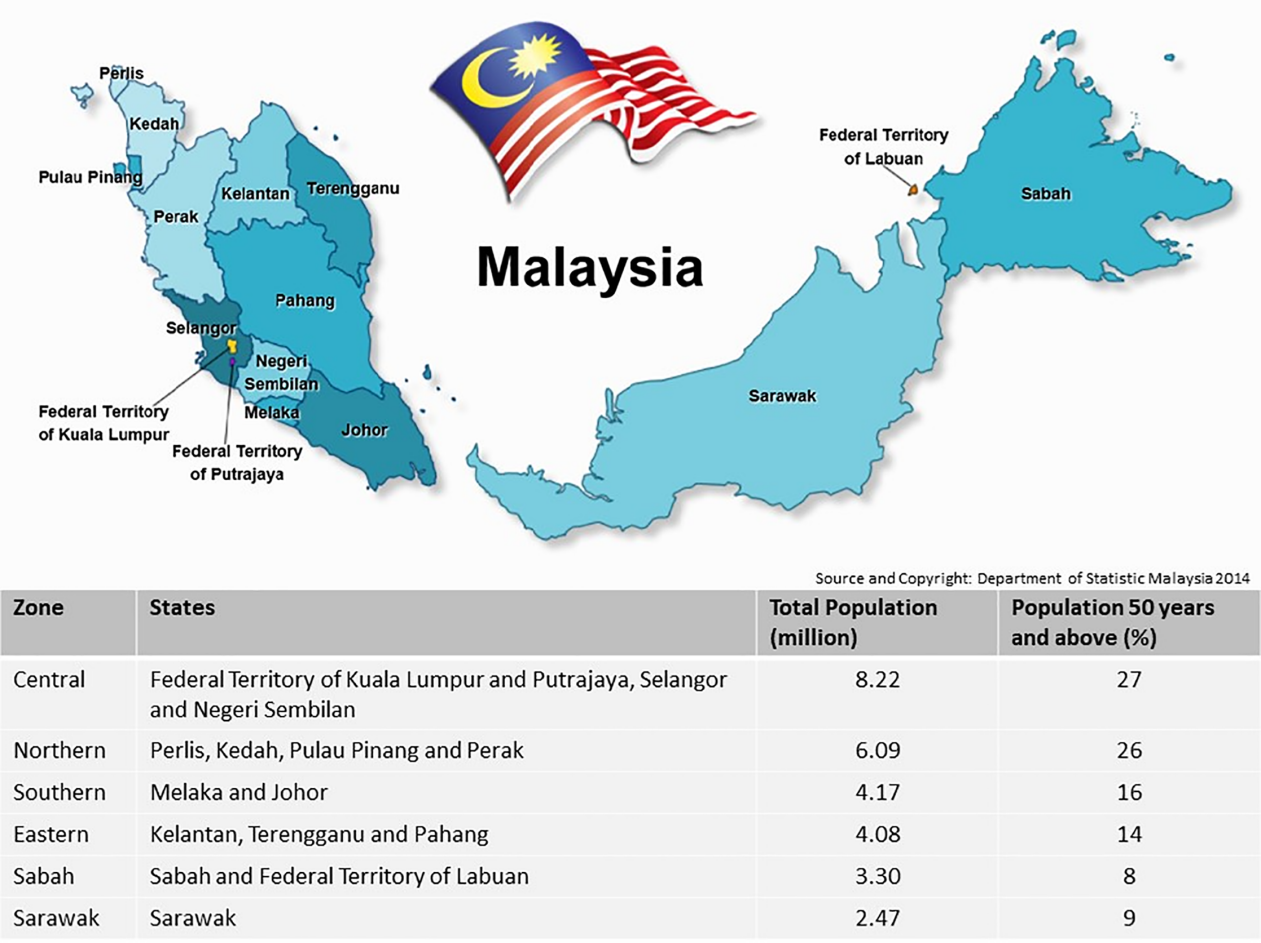
Population and states in Malaysia. Source: Department of Statistics, Malaysia.

This cross-sectional, population-based study was performed from 31^st^ September to 31^st^ November 2014. Ethical approval was obtained from the Medical Research and Ethics Committee of the Malaysian Ministry of Health. Malaysia was divided into six administrative regions for survey purpose; Northern (Perlis, Kedah, Pulau Pinang and Perak), Eastern (Kelantan, Terengganu and Pahang), Central (Wilayah Persekutuan Kuala Lumpur, Putrajaya, Selangor and Negeri Sembilan), Southern (Melaka and Johor), Sabah (State of Sabah and Wilayah Persekutuan Labuan) and Sarawak (Fig 1). Each individual region had 5 teams comprising of 3 persons, namely 1 medical officer and 2 paramedical staff (or 1 paramedical staff with 1 optometrist). Both doctors and paramedical staff in the team were trained in Ophthalmology.

Each team was responsible to survey 10 randomly selected blocks, examining 50 residents aged 50 years and above. The surveys in all regions were done simultaneously. A separate RAAB was conducted in each of these 6 regions.

Population sampling was in accordance to RAAB methodology, which is a widely used, World Health Organization (WHO)-recommended method for population-based survey of the prevalence of visual impairment and its causes [7]. The RAAB survey protocol and methodology has been described elsewhere [8,9].

### Sampling frame

An Enumeration Block (EB) is the smallest population unit, created by the national census office, to conduct the national population census once every ten years. The census office creates detailed geographical maps, indicating the exact location and boundaries of each EB in the country. The complete list of all EBs from the most recent national census was used to select clusters to be used in the RAAB. A total of 50 EBs (clusters) was randomly selected for each region, regardless of strata, using probability proportionate to size (PPS) technique. Individual EB codes were then used to identify location of the EBs and release of maps for fieldwork.

### Training

Training for survey teams was conducted prior to fieldwork in each region to ensure data quality and strict adherence to study protocol. Survey team members were required to attend four days of training, which included RAAB lectures, inter-observer variation assessment and fieldwork. Pilot surveys were done in the nearby selected EBs during fieldwork.

Each region had one coordinator who was responsible for the smooth implementation and progress of the survey. There were five survey teams in each region. The team members were all trained through RAAB training by a certified Western Pacific RAAB trainer (Dr Mohamad Aziz Salowi).

#### Survey methods

Each team was assigned to survey 10 EBs according to RAAB protocol.

Subjects were selected from each block using compact segment sampling method. The population area was divided into segments of equal population size, enough to provide the required number of eligible people aged 50 years and above. If the subject was not available at home, the subject’s contact number was taken from the neighbors and revisit would be done later. If after three revisits the subject could still not be examined, this person would be recorded as ‘Not available’ and the vision status as reported by relatives or neighbours would be taken.

Door-to-door interviews were conducted in each randomly selected EB. Subjects were recruited if they were aged 50 years and above and gave informed consent. A total of 50 subjects were recruited in each EB. All recruited subjects had a brief interview, where pertinent demographic details, medical and ocular history were noted. This was followed by visual acuity assessment with tumbling ‘E’ Snellen optotypes. Ocular examination was performed by medical officers using direct ophthalmoscope. The quality control measures for data collection included having pre-measured 6-meter ropes for each team, measuring visual acuity and pin hole with tumbling E chart repeatedly 5 times for every subject and having one-page data collection forms for each subject.

Should subjects have visual impairment, the primary cause of visual impairment was documented and the subjects were referred to the nearest ophthalmic care facility for further management. In cases of presenting VA<6/18 where the retinal status could not be determined, but the cornea, anterior segment and lens were clear, and there was no history of amblyopia or neurological disorders, then ‘other posterior segment disease’ had to be marked.

Inter-observer variation was conducted separately in each region prior to the survey. During the assessment in each region, each of the five survey teams examined the same 50 individuals. Findings of the other teams were compared to the findings of the most senior/experienced team (gold standard).

These subjects were patients from the ophthalmology outpatient department and staff. There were 15 to 20 people with impaired vision and at least 10 with cataract or (pseudo)aphakia or posterior segment diseases.

### Definition

This survey used WHO definition of blindness and visual impairment [10]. Blindness was defined as the presenting visual acuity of worse than 3/60 in the better eye. Severe visual impairment was defined as presenting visual acuity worse than 6/60 but better than or equal to 3/60 in the better eye. Moderate visual impairment was defined as presenting visual acuity worse than 6/18 but better than or equal to 6/60 in the better eye. Early visual impairment was defined as presenting VA<6/12 but better than or equal to 6/18 in the better eye. In all subjects with VA <6/12, a diagnosis of the primary cause of the visual impairment in the eye and in the person was given.

Avoidable blindness was specified as blindness that can be treated (cataract, uncorrected refractive errors, pterygium), or that could have been prevented by primary eye care (corneal opacity, phthisis) or by specialised ophthalmic services (cataract surgical complications, myopic degeneration, glaucoma, diabetic retinopathy).

Refractive error was determined as presenting distant VA<6/12, improving with pinhole to VA≥6/12.

### Sample size calculation

Population data was obtained from the Malaysian National Census 2010[11]. A prevalence of blindness of 3% in people aged 50 and above was assumed, based on experience from the previous National Eye Survey using a 95% confidence interval and a maximum variation around the estimate of 30% [5]. For each region, this resulted in a sample size of 50 clusters (2500 people aged 50 years and above) with an estimated design effect (DEFF) of 1.5.

### Data analysis

Each region had an appointed coordinator who was responsible for survey progress, data compilation and quality and other logistics such as financial support. Difficulties encountered were mainly due to geographical locations of certain blocks especially in Sabah and Sarawak.

RAAB used three levels of quality control. The first level was through validation rules incorporated into the RAAB software. Any errors or unlikely entries were shown in red with an explanatory text. The second level was by generating consistency reports in the RAAB software. Any errors listed would be corrected. The third level, to minimize data entry errors, was by validation of double data entry. Two different data entry personnel entered the same survey forms in two different data files. These two data files were then compared. If nothing was listed, the data files were identical and no data entry errors were made. If differences were detected, the corresponding survey record would be checked to find the correct entry. Both data files would be corrected until no errors were reported.

Survey team members entered collected data into specially designed RAAB 6 software. They performed consistency checks and validation through double data entry for all EB data. The processed data was emailed in specific folders to the regional coordinators who were responsible for merging the data files. Reports were automatically generated using the report generation module in the RAAB software. The findings of all regions were multiplied with a weighting factor for the population aged 50+ in each region to calculate the weighted prevalence of blindness and visual impairment for entire Malaysia.

Inter-observer variation was calculated for all zones surveyed and the Kappa coefficient values were classified as poor (<0.20), fair (0.21–0.40), moderate (0.41–0.60), good (0.61–0.80) and very good (0.81–1.00).

## Results

A total of 15,000 persons over the age of 50 years old (2,500 persons per region) were enumerated and 14,289 were examined (95.3% response rate). These subjects represented 0.26% of all people aged 50+ in the total region surveyed. Of the 711 non-respondents, 113 (0.8%) were not available, 443 (3.1%) refused to participate and 155 were not capable of being examined due to communication problems such as being deaf or demented (1.0%). The majority of the examined subjects were female (56.1%; 8,016) and this trend was seen in all regions. (Table 1).

**Table 1:** Survey area population.

	Northern		Eastern		Central		Southern		Sabah		Sarawak	
	Sample	Survey area	Sample	Survey area	Sample	Survey area	Sample	Survey area	Sample	Survey area	Sample	Survey area
	n (%)	n (%)	n (%)	n (%)	n (%)	n (%)	n (%)	n (%)	n (%)	n (%)	n (%)	n (%)
**Age group:**												
50–59 years	982 (40.6)	674,600 (47.1)	1,039 (42.4)	398,100 (50.3)	1,093 (47.9)	788,800 (53.6)	892 (37.6)	422,500 (49.9)	1,171 (49.1)	248,000 (57.8)	1,033 (43.3)	247,300 (49.4)
60–69 years	913 (37.8)	457,800 (31.9)	833 (34.0)	244,400 (30.9)	779 (34.1)	446,100 (30.3)	899 (37.9)	259,400 (30.6)	684 (28.7)	111,200 (25.9)	774 (32.5)	149,900 (29.9)
70–79 years	427 (17.7)	222,400 (15.5)	449 (18.3)	109,000 (13.8)	330 (14.5)	175,100 (11.9)	454 (19.1)	118,900 (14.0)	401 (16.8)	51,200 (11.9)	425 (17.8)	71,800 (14.3)
≥80 years	96 (4.0)	78,900 (5.5)	128 (5.2)	39,400 (5.0)	81 (3.5)	61,200 (4.2)	126 (5.3)	46,600 (5.5)	128 (5.4)	19,000 (4.4)	152 (6.4)	31,600 (6.3)
**Gender:**												
Men	1,031 (42.6)	698,000 (48.6)	1,014 (41.4)	384,900 (48.7)	1,006 (44.1)	752,200 (51.1)	1,061 (44.7)	429,200 (50.6)	1,065 (44.7)	227,700 (53.0)	1,096 (46.0)	260,000 (51.9)
Women	1,387 (57.4)	735,700 (51.3)	1,435 (58.6)	406,000 (51.3)	1,277 (55.9)	719,000 (48.9)	1,310 (55.3)	418,200 (49.4)	1,319 (55.3)	201,700 (47.0)	1,288 (54.0)	240,600 (48.1)
**Total**	**2,418**	**1,433,700**	**2,449**	**790,900**	**2,283**	**1,471,200**	**2,371**	**847,400**	**2,384**	**429,400**	**2,384**	**500,600**

Inter-observer variation calculated was classified as moderate with 78% of all zones surveyed having a kappa coefficient value of 0.41 or better. Zones with fair inter-observer agreement (Kappa coefficient of 0.21–0.40) comprised of 19% of the zones surveyed.

The age and gender-adjusted prevalence of bilateral blindness, severe VI and moderate VI was 1.2% (1.0–1.4), 0.9% (0.6–1.2) and 5.5% (4.9–6.1) respectively. Inter-region, Sabah had the highest prevalence of adjusted blindness and moderate and severe VI (at 1.9% and 9.4%, respectively. This was nearly four times the prevalence of blindness and more than twice the prevalence of moderate and severe VI, compared to the central region (0.5% and 4.6%, respectively). In this survey, there was no significant difference in prevalence of visual impairment and blindness between male and female subjects in the same region (Table 2)

**Table 2:** Distribution of PVA (presenting visual acuity) in the better eye in the sample population.

Region	Gender	N	Bilateral blindness (VA<3/60)		Bilateral severe VI (VA<6/60 and VA ≥3/60		Bilateral moderate VI (VA<6/18 and VA≥6/60)	
			n	Prevalence (95% CI)	n	Prevalence (95% CI)	n	Prevalence (95% CI)
Northern	Male	1,031	12	1.2 (0.6, 1.7)	12	1.2 (0.6, 1.7)	61	5.9 (4.3, 7.5)
	Female	1,387	25	1.8 (1.0, 2.6)	19	1.4 (0.7, 2.0)	91	6.6 (4.9, 8.2)
Eastern	Male	1,014	15	1.5 (0.8, 2.2)	12	1.2 (0.5, 1.9)	57	5.6 (4.2, 7.1)
	Female	1,435	25	1.7 (1.1, 2.4)	20	1.4 (0.8, 2.0)	90	6.3 (4.8, 7.7)
Central	Male	1,006	7	0.7 (0.2, 1.2)	5	0.5 (0.1, 0.9)	39	3.9 (2.6, 5.2)
	Female	1,277	3	0.2 (0.0, 0.5)	4	0.3 (0.0, 0.6)	61	4.8 (3.2, 6.4)
Southern	Male	1,061	11	1.0 (0.4, 1.7)	8	0.8 (0.3, 1.2)	55	5.2 (3.6, 6.8)
	Female	1,310	11	0.8 (0.3, 1.4)	11	0.8 (0.3, 1.4)	56	4.3 (3.0, 5.5)
Sabah	Male	1,065	23	2.2 (1.3, 3.0)	14	1.3 (0.6, 2.0)	106	10.0 (8.2, 11.7)
	Female	1,319	32	2.4 (1.6, 3.3)	22	1.7 (0.9, 2.4)	115	8.7 (7.1, 10.3)
Sarawak	Male	1,096	13	1.2 (0.6, 1.8)	12	1.1 (0.5, 1.7)	94	8.6 (6.6, 10.5)
	Female	1,288	28	2.2 (1.4, 3.0)	17	1.3 (0.6, 2.1)	104	8.1 (6.0, 10.1)
Malaysia	Male	6,273	81	1.2 (0.9, 1.5)	63	0.9 (0.7, 1.1)	412	5.8 (5.1, 6.5)
	Female	8,016	124	1.3 (1.0, 1.6)	93	1.0 (0.8, 1.2)	517	6.0 (5.3, 6.7)

VI, visual impairment; VA, visual acuity; CI, confidence interval

The commonest causes of blindness were untreated cataract (58.6%), diabetic retinopathy (10.4%), other posterior segment disease (8.4%) and glaucoma (6.6%). Untreated cataract (68.0%), uncorrected refractive error (14.4%) and diabetic retinopathy (6.1%) represented the commonest causes of moderate and severe VI. Overall, 86.3% of the causes of blindness were avoidable and 58.6% of the causes of blindness were treatable. Avoidable causes of moderate and severe VI combined represented 96.3% of the study population and treatable causes of low vision 82.4% (Table 3).

**Table 3:** Principal causes of blindness and visual impairment by person.

**Primary cause of VI (VA<6/18)**	**Blindness**		**Severe VI**		**Moderate VI**	
	**n**	**%**	**n**	**%**	**n**	**%**
1. Uncorrected refractive error	0	0.0	4	4.1	131	15.9
2. Aphakia uncorrected	0	0.0	0	0.0	2	0.2
3. Cataract untreated	133	58.6	116	70.1	688	68.2
4. Cataract surgical complication	6	5.3	2	1.1	34	4.6
5. Pterygium	4	0.7	2	0.9	4	0.2
6. Corneal opacity	8	3.5	3	1.4	1	0.3
7. Pthisis	3	0.7	0	0.0	0	0.0
8. Myopic Degeneration	1	0.4	0	0.0	1	0.3
9. Glaucoma	14	6.6	6	4.4	14	2.0
10. Diabetic Retinopathy	9	10.4	13	8.4	33	5.4
11. ARMD	0	0	1	0.8	4	0.6
12. Other Posterior Segment Disease	18	8.4	7	7.0	11	1.5
13. All Other Globe/CNS Abnormalities	9	5.4	2	1.7	6	0.8
Total	205	100.0	156	100.0	929	100.0
**Intervention Category**	**Blindness**		**Severe VI**		**Moderate VI**	
	**n**	**%**	**n**	**%**	**n**	**%**
A. Treatable (1,2,3)	133	58.6	120	74.2	821	84.3
B. Preventable (PHC/PEC Services) (5,6,7,8)	16	5.3	5	2.3	6	0.8
C. Preventable (Ophthalmic Services) (4,9,10)	29	22.3	21	13.9	81	12.0
D. Avoidable (A+B+C)	178	86.2	146	90.4	908	97.1
E. Posterior Segment Causes (8,9,10,11,12)	42	25.8	27	20.6	63	9.8

VI, visual impairment; VA, visual acuity; ARMD, age-related macula degeneration; CNS, central nervous system; PHC, primary health care; PEC, primary eye care

Cataract surgical coverage (CSC) was defined as the number of people in the survey who had cataract surgery compared to the number who required surgery [9]. This was calculated for eyes as well as for persons. The weighted average CSC for blindness, severe visual impairment and moderate visual impairment in persons was 90%, 86% and 66% respectively. Male subjects had slightly higher CSC at all levels of visual acuity. The overall CSC for blind and severely visual impaired eyes was fairly high (77% and 73% respectively). Effective cataract surgical coverage (eCSC) is a relatively new indicator, measuring the proportion of people in the sample who were operated in one or both eyes for cataract and achieved a presenting VA of 6/18 or better, out of all people with bilateral cataract (Table 4).

**Table 4:** Cataract surgical coverage (CSC) by person and eyes for sample population.

		CSC (eyes)—%e			CSC (persons)—%			eCSC (persons)—%		
		VA < 3/60	VA < 6/60	VA < 6/18	VA < 3/60	VA < 6/60	VA < 6/18	VA < 3/60	VA < 6/60	VA < 6/18
**Central**	**Males**	90.7	88.0	75.1	100.0	95.3	84.2	93.8	89.4	78.2
	**Females**	92.4	91.3	72.9	100.0	100.0	83.0	93.6	93.6	76.1
	**Total**	91.7	89.9	73.8	100.0	98.1	83.5	93.7	91.9	76.9
**Eastern**	**Males**	72.7	65.2	40.9	97.1	83.7	55.6	85.7	72.1	44.4
	**Females**	66.0	58.8	36.4	81.7	74.4	50.7	71.8	66.3	43.9
	**Total**	68.5	61.1	38.0	86.8	77.5	52.3	76.4	68.2	44.1
**Northern**	**Males**	73.2	69.9	52.0	89.0	85.9	67.3	80.8	78.2	60.9
	**Females**	75.0	70.8	55.5	89.1	86.8	71.4	82.8	80.9	66.3
	**Total**	74.3	70.5	54.1	89.1	86.5	69.8	82.1	79.9	64.2
**Sabah**	**Males**	58.3	52.7	30.7	77.6	70.9	40.2	61.2	56.4	32.4
	**Females**	49.7	41.7	24.0	61.7	54.3	32.1	55.0	48.6	27.7
	**Total**	53.6	46.5	26.8	68.8	61.6	35.6	57.8	52.0	29.7
**Sarawak**	**Males**	70.8	65.1	34.4	94.1	87.3	44.3	80.4	74.6	37.7
	**Females**	65.4	60.2	32.6	77.9	75.3	42.3	69.1	67.1	36.5
	**Total**	67.8	62.3	33.4	84.9	80.5	43.2	74.0	70.3	37.1
**Southern**	**Males**	76.0	73.0	55.6	94.2	92.5	74.3	80.8	79.3	63.5
	**Females**	82.6	79.8	61.3	92.9	89.8	72.3	84.5	80.7	64.7
	**Total**	80.0	77.1	59.0	93.4	90.8	73.1	83.1	80.1	64.3
**Malaysia**	**Males**	73.6	69.0	48.1	92.0	85.9	61.0	80.5	75.0	52.9
	**Females**	71.9	67.1	47.1	83.9	80.1	58.6	76.1	72.9	52.6
	**Total**	72.6	67.9	47.5	87.2	82.5	59.6	77.8	73.7	52.7

VA, visual acuity

In terms of barriers to cataract surgery, a third of the subjects felt they did not need surgery (33.2%). ‘Fear of undergoing surgery or poor outcome’ (22.9%), ‘local reasons’ such as long waiting time for appointments at local hospitals and transportation issues (20.6%) and ‘cost of surgery’ (9.5%) were other reasons for not seeking cataract surgery. The central region, being considered as the urban region of Malaysia showed easy accessibility of eye care services, high awareness of importance of cataract surgery and good purchasing power for surgical needs. On the other hand, Sabah and Sarawak had the poorest accessibility (13.4% and 19.5% respectively), caused by the geographical terrain of these two regions (Table 5).

**Table 5:** Patient perceived barriers to cataract surgery.

	Northern		Eastern		Central		Southern		Sabah		Sarawak		Malaysia	
	n	%	n	%	n	%	n	%	n	%	n	%	n	%
No Need	18	47.4	13	32.5	2	40.0	4	20.0	8	11.9	6	14.6	51	33.2
Fear	4	10.5	9	22.5	2	40.0	4	20.0	14	20.9	6	14.6	39	22.9
Cost	2	5.3	3	7.5	0	0.0	4	20.0	23	34.3	4	9.8	36	9.5
Denied	2	5.3	1	2.5	0	0.0	0	0.0	0	0.0	1	2.4	4	1.9
Unaware	4	10.5	4	10.0	0	0.0	2	10.0	5	7.5	3	7.3	18	7.0
No Access	1	2.6	4	10.0	0	0.0	0	0.0	9	13.4	8	19.5	22	4.9
Local	7	18.4	6	15.0	1	20.0	6	30.0	8	11.9	13	31.7	41	20.6

## Discussion

The population of elderly in Malaysia is expected to rise further and with it, the burden of age-related eye diseases such as cataract, diabetic retinopathy and glaucoma. The life expectancy at birth for 2017 in Malaysia is 74.8 years. [12]. This is expected to increase to 77.6 years by 2030. [13]. The NES II was the first RAAB survey in Malaysia and represented the country’s commitment towards the WHO Global Action Plan for the Prevention of Avoidable Blindness and Visual Impairment with a special focus towards elderly patients.

NES I conducted in 1996 comprised of subjects of all ages living in randomized living quarters. The blocks were randomized through multistage sampling method [5]. Due to different methodology and subject ages, our study outcomes were not comparable to NES 1. Earlier studies indicated that 80–90% of all blindness occurred in people aged 50 and above and that the causes of blindness in this age group were a good indicator for those in the entire population [14].

Untreated cataract, which was reported to be the leading cause of blindness (39%) in 1996, remained the leading cause of blindness in our study [5]. Uncorrected refractive error, which was noted to be the main cause of low vision in the NES I was replaced by cataract in our study [5]. NES 1 only reported overall country prevalence of visual impairment. In comparison to NES 1, our study had an advantage of giving the prevalence of visual impairment of different regions in addition to the overall country prevalence [5].

Our study prevalence of bilateral blindness of 1.2% was consistent with reported RAAB studies that reported the prevalence of blindness to range from 1.2% to 4.4% [15,16]. We noted our prevalence of moderate VI of 5.9% and severe VI of 1.0%, corresponded to other studies, which reported the prevalence of moderate VI to range from 4.1% to 16.8% and severe VI to range from 1.1% to 4.4% [17–19]. Sabah and Sarawak had the highest prevalence of visual impairment as these two regions had the poorest access to ophthalmological services.

The age-standardized prevalence of cataract and uncorrected refractive error from the year 1990 to 2013 has been reported to decrease by 17.6% and 5.2% respectively. [20]^.^ Our study noted there was no blindness due to refractive error and refractive error was not a major cause of moderate VI. This may be because our study collected data on presenting VA rather than unaided VA. Despite advances in delivery of eye care services, cataract remains the leading cause of blindness in developing countries, accounting for 50% of the causes of blindness [21]. This was confirmed in our study where untreated cataract was the commonest cause of blindness and moderate and severe VI.

Cataract surgical services in Malaysia has expanded more than 2-fold, with the number of public hospitals performing more than 1000 cataract surgeries per year increasing from 4 in the year 2002 to 10 in the year 2011 [22]. A global survey of cataract surgical coverage noted that the CSC (persons) rate ranged from 29% to 92.8% with females getting less access to cataract surgery compared to males [23]. Our CSC of 66% to 90% was in concordance to reported literature.

Treatment of cataract involves surgical intervention and it has been shown that apart from accessibility and financial barriers, social factors such as low awareness, fear and cultural beliefs towards cataract surgery also influence the surgical coverage outcome [24]. This was reinforced in our study where our cataract surgical coverage was less than 70% for subjects with moderate visual impairment, and social factors represented 83.7% of the reasons patients refused cataract surgery.

Limitations of this study were those weaknesses inherent to RAAB methodology such as the inability to validate patient responses as patients were seen only once and diagnostic limitations as portable instruments were used. This study however is still valid as it is the first elderly-focused, large population-based study with inter-regional sampling in Malaysia. The high response rate of 95.3%, standardized protocol and experienced personnel further strengthened our study methodology. In our view RAAB is more time-efficient compared to conventional sampling methods, the RAAB method is also able to assess effectiveness of interventional activities more efficiently so adjustments can be made for future prevention of blindness programs.

In summary, the high level of avoidable and treatable blindness in our study indicated a need to further expand and improve ophthalmological services in Malaysia, especially in the regions Sabah and Sarawak. Efforts also need to be stepped up for increased patient education and awareness of ocular health to reduce the incidence of cataract surgery refusal from social causes.

## Supporting information

S1 Appendix NES II survey forms. (PDF)
